# Evolution of the Viscoelastic Properties of Filler Reinforced Rubber under Physical Aging at Room Temperature

**DOI:** 10.3390/polym15071806

**Published:** 2023-04-06

**Authors:** María Vizcaíno-Vergara, Leif Kari, Lewis B. Tunnicliffe, James J. C. Busfield

**Affiliations:** 1 The Marcus Wallenberg Laboratory for Sound and Vibration Research (MWL), Department of Engineering Mechanics, KTH Royal Institute of Technology, Teknikringen 8, 114 28 Stockholm, Sweden; 2 School of Engineering and Materials Science, Queen Mary University of London, Mile End Road, London E1 4NS, UK; 3 Birla Carbon, 1800 West Oak Commons Court, Marietta, GA 30062-2253, USA

**Keywords:** physical aging, carbon black, natural rubber, reinforced rubber, torsion pendulum, viscoelastic properties, storage modulus, loss modulus, external surface area, compressed oil adsorption number

## Abstract

Filler reinforced rubber is widely used for engineering applications; therefore, a sound characterization of the effects of physical aging is crucial for accurately predicting its viscoelastic properties within its operational temperature range. Here, the torsion pendulum is used to monitor the evolution of the storage and loss modulus of carbon black filled samples for four days after a temperature drop to 30 °C. The storage modulus presents a continuous increase, while the loss modulus generally displays a steady decrease throughout the four days that each test was conducted. The relationship of the recovery rates with the carbon black properties is also studied, analysing its dependency on the particle size and aggregate structure. The evolution of the recovery rate seems to depend linearly on the surface area while the carbon black structure appears to have a much weaker influence on the physical aging behavior for the set of compounds tested. The obtained results corroborate the presence of physical aging at room temperature for filler rubber materials and the ability of the torsion pendulum to monitor the storage and loss modulus change, providing pivotal data on the influence of physical aging on the viscoelastic properties of the material.

## 1. Introduction

Filler-reinforced rubber materials are widely used in engineering applications due to their high stiffness, abrasion resistance, and hardness when compared to unfilled rubber [1]. Because of its widespread usage, lots of efforts have been made in the development of constitutive models that are able to capture the different dependencies displayed by filled rubber on the strain amplitude, magnetic field, frequency, temperature and chemical aging [2,3,4,5,6,7,8,9,10,11]. However, the effect of physical aging on the viscoelastic properties has been less widely studied, with just a few authors attempting to evaluate this dependency [12,13,14].

**Figure 1:**
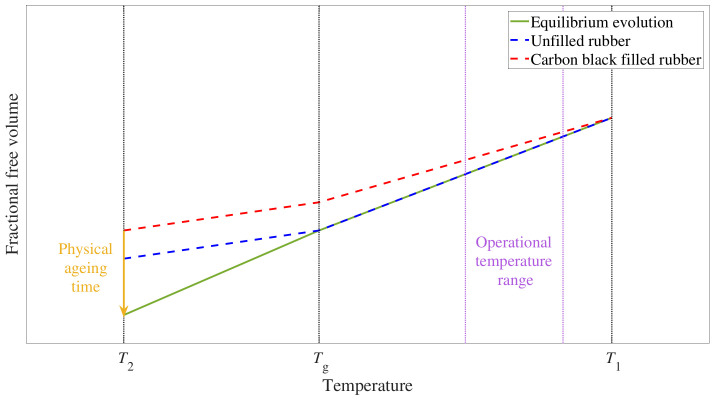
Schematic representation of the free volume evolution after a temperature change from $T_{1}$ to $T_{2}$, below $T_{g}$. The equilibrium evolution is plotted as a solid green line, the evolution for unfilled rubber is plotted as a dashed blue line and the evolution for filled rubber is plotted as a dashed red line. The violet band indicates the operational temperature of rubber for a wide variety of applications, where a deviation from equilibrium is observed for filler reinforced rubber.

Physical aging refers to the process by which the material tends to recover its thermodynamic equilibrium after a temperature change [15]. This phenomenon is schematically depicted in Figure 1, with the evolution of free volume against temperature. A decrease in temperature leads to a reduction of free volume, which instantaneously reaches its equilibrium value when the temperature change is restricted to temperatures above the glass transition temperature, $T_{g}$. Conversely, for temperature changes below $T_{g}$, the slower chain dynamics result in longer times needed to reach the equilibrium free volume value at the final temperature [16]. During this time, the continuous change of free volume toward equilibrium leads to shifting mechanical properties and it is therefore called physical aging time. As shown in Figure 1, for unfilled rubber (blue line), this deviation from equilibrium (green line) only happens below $T_{g}$. This low-temperature range is not the operational temperature of most rubber materials due to their brittle behavior [17,18], which may explain why this phenomenon has not been frequently included in the aforementioned constitutive models.

However, recent experimental findings on filler network dynamics at small strains [19] suggested physical aging happening also above the glass transition temperature for carbon black filled reinforced rubbers. This different evolution for filled rubber is depicted with a red line in Figure 1, showing how additional times would be needed to reach the equilibrium free volume, and consequently the final properties, at temperatures well above $T_{g}$. This is of great consequence since most carbon black filled rubber applications, from tires to vibration isolators including bearings and O-rings, operate at room temperature or above. Therefore, a deeper understanding of the effect of physical aging on the viscoelastic properties of filled rubber is needed for an accurate prediction of its mechanical performance and service life [20].

This work focuses on the experimental measurement of the evolution of the viscoelastic properties under physical aging conditions. To trigger the physical aging process, the sample is submitted to a rapid temperature change [16], and its storage and loss moduli are measured with the torsion pendulum. The obtained results render additional data on the evolution of the viscoelastic properties of carbon black filled rubber with physical aging time and their relationship with the carbon black surface and structure characteristics. Therefore, they provide not only a deeper insight into the effect of physical aging on the macroscopic properties of the material but also the means to better understand the process itself.

The structure of this work is divided into four sections. In Section 2, the equipment and experimental steps followed to measure physical aging are introduced. Next, Section 3 presents the evolution obtained for the modulus, showing an increase in the storage modulus and a decrease in the loss modulus with physical aging time for most of the tested samples. Additionally, the relationship of the physical aging recovery rate with the carbon black characteristics is studied to help understand the different factors influencing the physical aging evolution. Finally, Section 4 gives a summary of the results and the future steps.

## 2. Method

In order to analyze the effect of physical aging on the viscoelastic properties, the storage and loss modulus of the samples are monitored for an extended physical aging time of up to four days after a temperature drop until they close to the reach of equilibrium.

### 2.1. Equipment

**Figure A1:**
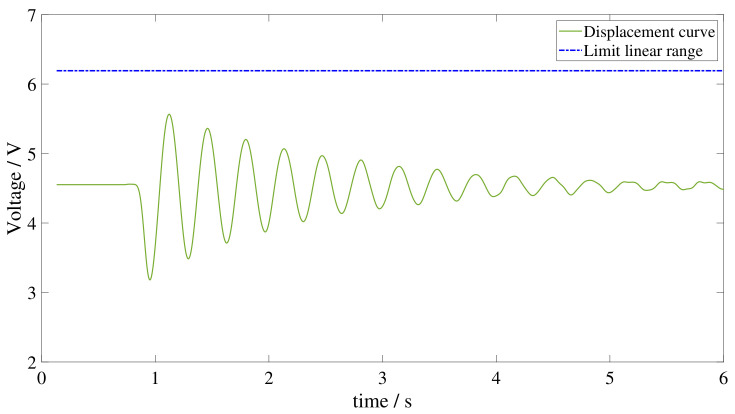
Resulting exponentially decreasing sinusoidal curve (green) and limit for the linear viscoelastic regime (blue). Adapted from Vizcaíno-Vergara et al. [31].

The torsion pendulum is used to monitor the properties evolution at small strains due to its versatility and easy customization [21,22]. Since its appearance more than two centuries ago [23], the torsion pendulum has been extensively used for material characterization [24,25,26,27], and its specific applicability to rubber and filler reinforced rubber systems has been previously established [19,28,29,30,31]. Furthermore, the torsion pendulum allows for long testing times, a crucial requirement for physical aging measurements [16]. The torsion pendulum is based on the free vibration principle, in which the sample is perturbed to initiate a rotational (twisting shear) motion and then left to freely vibrate until repose [32]. The displacement of the sample is measured and the shape of the resulting curve is schematically presented in Figure A1 in Appendix A.

**Figure 2:**
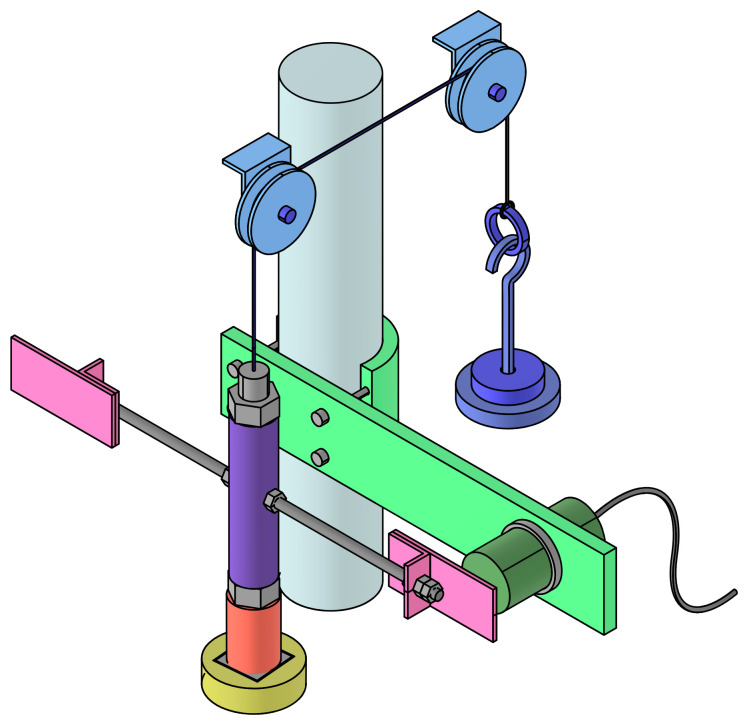
Schematic representation of the rubber sample (colored in orange) placed on the torsion pendulum where the electromagnet (yellow), inertial element (violet), counterbalance (dark blue), lateral plates (pink), and sensor (dark green) are shown. The complete setup and guidelines description can be found in Vizcaíno-Vergara et al. [31].

Figure 2 shows a representation of the sample set up in the torsion pendulum. An electromagnet (yellow) acts like the bottom clamp of the rubber cylinder (orange). Its upper fixture is obtained by hanging the sample from the inertial element (violet) which is, in turn, connected to a counterbalance (dark blue). Two simultaneous flows of pressurized air impacting the lateral plates (pink) are responsible for the initiation of the motion and the sensor (dark green) placed in front records the sample movement.

Once the rotational curves are obtained, a series of steps are followed to calculate the dynamic properties of the material. They are described in detail in the work by Vizcaíno-Vergara et al. [31] which is briefly mentioned here for clarity. First, the curves are filtered and the maxima and minima of the different peaks are identified. These points are then used for the calculation of the frequency, ω, and logarithmic decrement, Δ, of the curve. The testing conditions and the sample geometry may lead to the presence of additional frequencies different from the rotational one, which may hinder the calculus of accurate results. Therefore, a thorough identification of the frequencies present in the displacement curve is needed. For this analysis, Fourier Transform, FFT, is performed on the curve to extract its frequency components, and the fitting quality of the excitation curve to the ideally exponentially decreasing sinusoidal curve is also evaluated. Next, based on these FFT and fitting results, only the purely rotational curves are selected and used for the properties calculation. Finally, the elastic storage modulus, $G^{{}^{\prime }}$, and the loss shear modulus, $G^{\prime \prime }$ can then be obtained as [33]

(1) $$G^{\prime }=\frac{2}{\pi r^{4}}I\omega^{2}l$$

and

(2) $$G^{\prime \prime }=\frac{2}{r^{4}}\frac{I\omega^{2}\Delta }{\pi^{2}}l,$$

where *I* is the inertia of the inertial element, *l* the sample length and *r* the radius of the rubber cylinder.

At each measuring time, a minimum of fifteen excitations are performed on the sample, resulting in at least fifteen sinusoidal curves that can be used for the properties calculation. Each loss and storage modulus value is then calculated as the average of ten points, extracted from at least four different optimal excitations selected amongst the fifteen available ones.

### 2.2. Materials

The samples tested are cylindrical samples of diameter 12 mm and height 75 mm, and the material used is natural rubber filled with 50 parts per hundred (phr) of carbon black. The carbon black types used are N220, N326, N330, N339, N550 and N772 [34] and the material formulation is specified in Table 1. The compounds were provided by Birla Carbon (Birla Carbon, Marietta, GA 30062, USA) and the cured samples were obtained by compression molding at 150 °C for a total time of 33 min ($T_{90}+5\;\min$) as obtained by moving die rheometer (MDR) testing.

### 2.3. Experimental Procedure

In order to capture the evolution of the viscoelastic properties during the physical aging evolution, the rubber sample was submitted to a temperature change. After the temperature drop, the storage and loss modulus were measured at different physical aging times up to 96 h. This procedure allows for the evaluation of physical aging through its effect on the material properties instead of via the measurement of the free volume present in the structure [35,36]. The tests were conducted in the linear viscoelastic strain regime (γ<0.1%), to avoid any additional phenomena present at larger strains [37,38,39,40], and to isolate the effect of physical aging on the viscoelastic properties.

**Figure 3:**
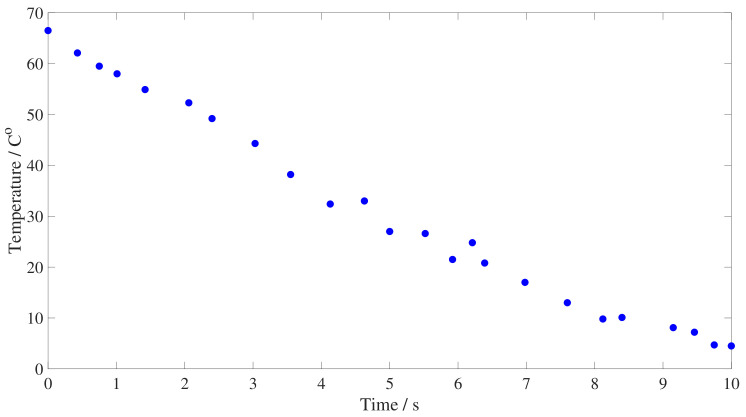
Set of points corresponding to the temperature of the sample after its submersion in liquid nitrogen for different amounts of time, from 0 to 10 s.

Besides the strain level, there are two parameters to be defined for the experimental characterization, the initial and final temperatures, and the temperature change rate. Firstly, both initial and final temperatures need to be well above the glass transition temperature, since our goal is to characterize physical aging at room temperatures for filler reinforced rubber [30]. This restricts the final temperature to be above $T_{\mathrm{room}}\approx 25\;{}^{\circ}C$, and because of the practicalities of an easier control of the temperature inside the oven chamber, the minimum final temperature of the test was further restricted to be above 30 °C. Additionally, the magnitude of the temperature change needs to be sufficient to lead to measurable physical aging effects but not too large that equilibrium can not be reached within the measured time frame, rendering the experimental characterization unfeasible. Considering previous data on the evolution of the mechanical properties with temperature [31], a temperature drop of at least 40 °C was selected for this experimental procedure. Based on these conditions, the initial temperature was set to 70 °C and the final temperature to 30 °C. It would be expected that variations of initial and final temperature within the prescribed requirements would also trigger the physical aging process although they are not explored in the current investigation. Secondly, the temperature change rate needs to be decided. Considering that the obtained physical aging data may be used, amongst other applications, for the development of a constitutive model, the temperature change rate was selected to be compatible with existing mathematical expressions of the physical aging evolution [14,41], leading to a steplike temperature change. In order to achieve this effect during testing, the sample was crash-cooled by submersion inside liquid nitrogen until the required final temperature was reached. The time needed for the desired temperature drop was obtained by measuring the final temperature in the core of a sample after submerging it in liquid nitrogen during increasing time intervals, from 0 to 10 s and starting from an initial temperature of 70 °C. The sample was then placed inside an oven at 30 °C, and its temperature was measured after 20 s, to allow for temperature stabilization across the sample. The sacrificial sample used for calibration was of the same dimensions and material as those used for the physical aging characterization since different materials and geometries would lead to different submersion times. The results, presented in Figure 3, show that the 12×75 mm sample loaded with 50 phr carbon black under study needs to be submerged in liquid nitrogen for 5 s to achieve a temperature drop from 70 to 30 °C.

Following these considerations, the temperature profile of each physical aging experiment consisted of two intervals. The preconditioning step started at room temperature of approximately 25 °C, then the temperature was increased to 70 °C, at which the sample was then annealed for 2 h. Next, the sample was crash cooled to 30 °C by submersion in liquid nitrogen for 5 s. The time when the sample was extracted from the liquid nitrogen was considered to be the start of the physical aging time. Following, the sample was placed inside a second oven, at a constant temperature of 30 °C, where the rest of the experiment took place.

**Figure 4:**
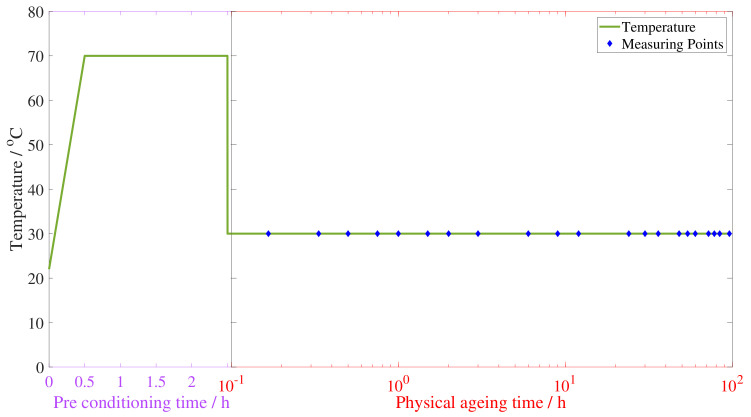
Temperature profile of the experimental procedure followed for each sample. The green line represents the temperature increase, annealing, crash cooling, and constant value until the end of the experiment. The blue diamonds mark the measurement times, the violet axis shows the preconditioning time in a linear time scale, and the red axis uses a logarithmic time scale to represent the physical aging time.

Finally, the measurements were performed following the torsion pendulum guidelines. The total measuring time was set to 96 h, to allow for a sufficient stabilization of the viscoelastic properties. The spacing of the measurements throughout the 96 h was defined logarithmically, to be able to capture the initial rapid evolution and to monitor the slow final advance toward equilibrium. The measurement times are 10, 20, 30, 45, 60, and 90 min; and 2, 3, 6, 9, 12, 24, 30, 36, 48, 54, 72, and 96 h. The list is included in Table A1 in Appendix A and the complete temperature profile is plotted in Figure 4.

## 3. Results and Discussion

### 3.1. Torsion Pendulum Results

**Figure 5:**
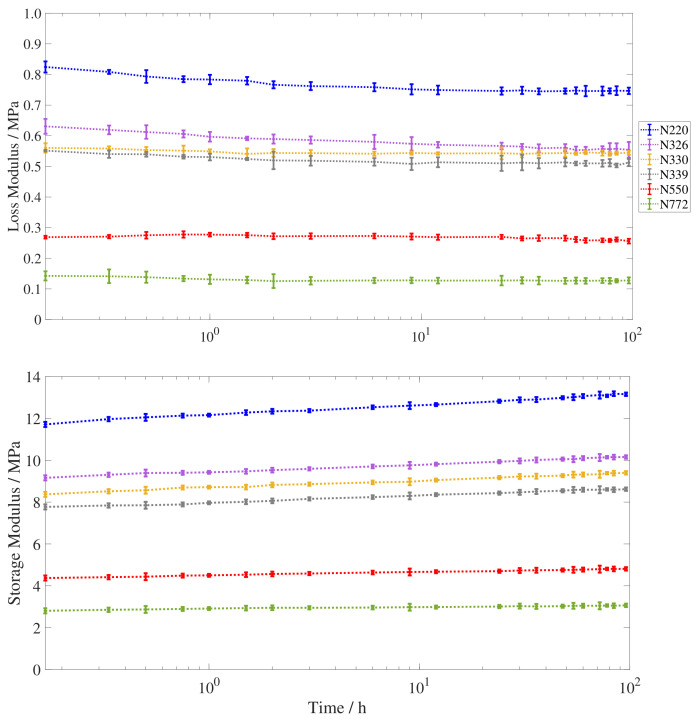
Evolution of the loss (top) and storage (bottom) modulus from 10 min to 96 h after a temperature drop from 70 to 30 °C. The different carbon black types are plotted in blue (N220), purple (N326), yellow (N330), grey (N339), red (N550), and green (N772).

The results for the storage and loss modulus are shown in Figure 5. They are obtained at the measuring times specified in Table A1 and the value at each time stamp is obtained as the average of ten points extracted from at least four different excitations of the torsion pendulum. The dashed line joins the average values and the deviation is represented by the T symbols at each time point.

The storage modulus presents a monotonic increase with physical aging time. This is the expected evolution as the sample is submitted to a temperature decrease [31,42]. Immediately after the temperature drop, the storage modulus increases but does not reach the final value instantaneously, instead, it continues to increase throughout the ninety-six following hours. The largest change is shown by the N220 sample with a 1.44 MPa increase, representing 12.3% of its value after the temperature drop. This increment is calculated between the value at 10 min (11.71±0.12 MPa) and the value at 96 h (13.15±0.08 MPa). The smallest increase is displayed by the N772 sample, with a 0.26 MPa increase, between its initial (2.81±0.04 MPa) and final (3.07±0.04 MPa) values. The calculated shift represents 9.3% of its 10 min value.

The loss modulus shows a monotonic decrease with physical aging time for all samples except for N550, for which it shows a slight increase during the first 45 min to later exhibit the monotonic decrease shown by the rest of the carbon black compounds. The largest decrease in loss modulus is shown again by sample N220, with a maximum difference of 0.078 MPa, between its value at 10 min (0.825±0.018 MPa) and its value at 96 h (0.747±0.011 MPa). This represents a decrease of 9.4% from the initial loss modulus immediately after the temperature change. N772 shows also the smallest difference between its initial and final values at 10 min (0.143±0.005 MPa) and 96 h (0.127±0.008 MPa) respectively. This difference is 0.016 MPa, which amounts to 10.9% of its initial measured value.

Additional Dynamic Mechanic Analysis (DMA) data for the evolution of the viscoelastic properties during physical aging can be found in Appendix B. The tendencies displayed by the modulus of two carbon black samples (N550 and N115) after a different temperature drop from 70 to 20 °C show an increase of the storage modulus and a decrease of the loss modulus with physical aging time. Despite the difficulty of quantitatively comparing the two procedures because of their different thermal histories, both DMA and torsion pendulum results display the same tendencies for the viscoelastic properties with physical aging time.

In a linear time scale, the time evolution of the storage modulus recovery presents a similar trend to that of the results obtained for thermal annealing of a CB-filled solution styrene-rubber [43] and preshearing [19], showing a combination of fast and slow recovery contributions. Intriguingly, the loss modulus shows a decreasing trend contrasting with the measured evolution for unfilled rubber after a temperature decrease [42]. This different evolution may be linked to the dual contribution of free volume and configurational changes to the global thermal equilibrium [44] and their contrasting effect on the mechanical properties via different equilibrium time scales. This could lead to multiple relaxation times for the volumetric, enthalpic, and mechanical equilibria, as previously discussed in the literature [45,46,47,48]. The existence of an initial rapid change followed by a slow stabilization may also help explain the initial overshoot observed for the loss modulus evolution of the N550 compound.

### 3.2. Carbon Black Properties Analysis

**Figure 6:**
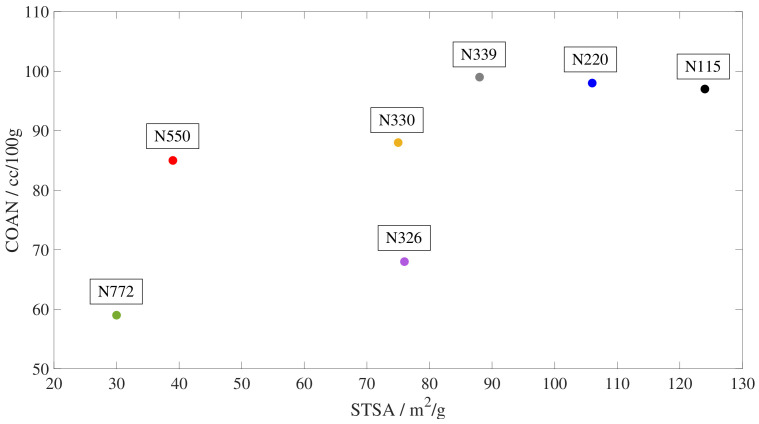
Colloidal plot showing the tested carbon black types. The abscissa represents the external surface area (STSA) and the ordinate represents the oil absorption number of the compressed sample (COAN).

**Figure 7:**
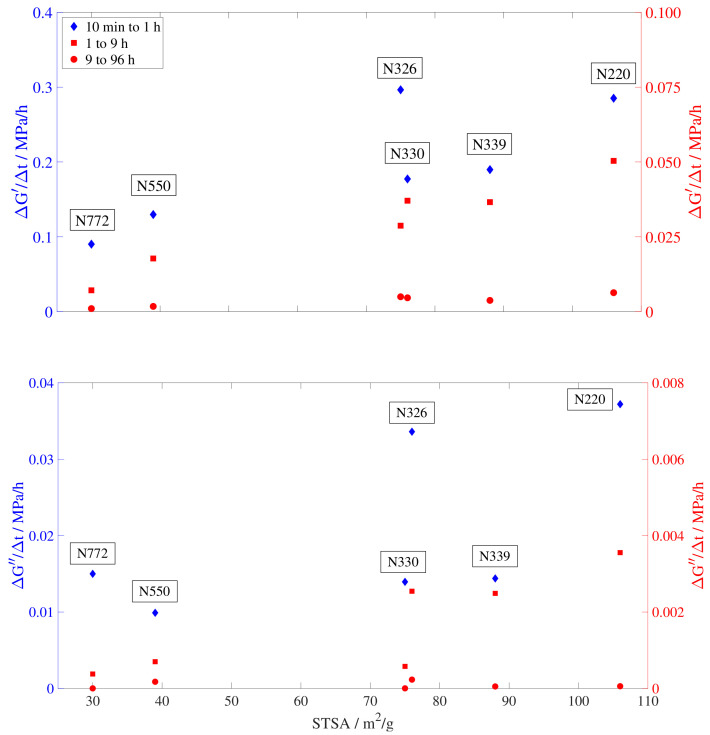
Dependency of the storage and loss modulus slope on the external surface area (STSA). Slopes are calculated from 10 min to 1 h (blue diamond), 1 to 9 h (red square), and 9 to 96 h (red circle).

**Figure 8:**
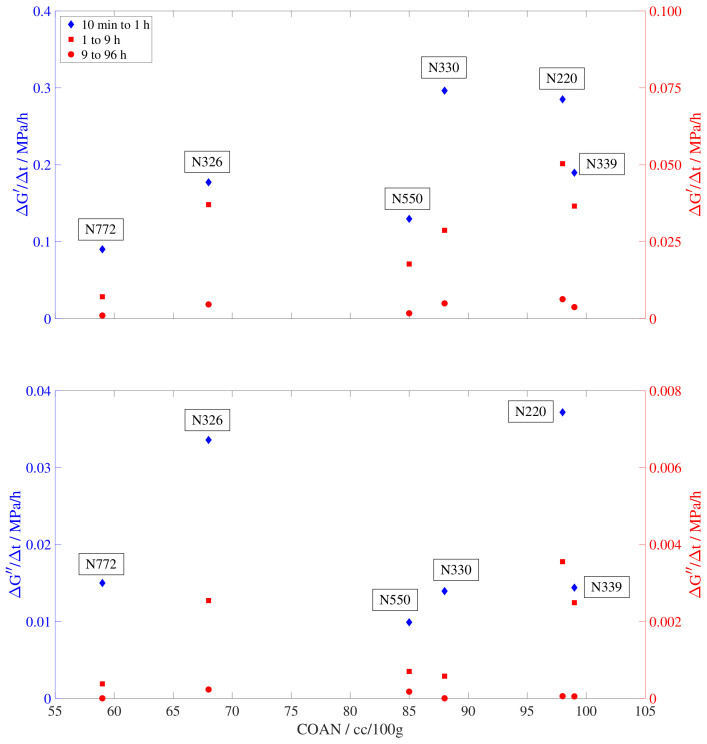
Dependency of the storage and loss modulus slope on the oil absorption number of the compressed sample (COAN). Slopes are calculated from 10 min to 1 h (blue diamond), 1 to 9 h (red square), and 9 to 96 h (red circle).

To throw some light on the mechanisms responsible for the different evolution of the storage and loss modulus, the slopes of the curves, $\Delta G^{{}^{\prime }}/\Delta t$ and $\Delta G^{\prime \prime }/\Delta t$, are plotted against the external surface area (STSA [m^2^/g]) and the oil absorption number of the compressed sample (COAN, [cc/100 g]). For clarity, the position in the colloidal plot of all tested samples is shown in Figure 6 and the results are presented in Figure 7 and Figure 8. Since the evolution of the storage and loss modulus presents a linear increase in a logarithmic time scale, three slopes are calculated to account for the different rate regimes. The first slope accounts for the increase rate immediately after the temperature change and it is calculated based on the values from 10 min to 1 h. The second slope reflects the intermediate times, from 1 to 9 h. Finally, the third rate is calculated based on the times required to approach equilibrium, from 9 to 96 h.

It can be seen from Figure 7 that both the storage and loss modulus slopes present a dependence on the external surface area. The slight deviation of the first slope (10 min to 1 h) is not unexpected due to the rapid changes in the storage and loss modulus during the initial moments after crash cooling. The tendencies of the other two slopes follow a linear increase for both storage and loss modulus versus STSA. This dependency on the STSA seems to indicate that those compounds with smaller carbon black particle sizes (N220, N326, N330, and N339) achieve their final properties faster than those with larger particle sizes (N550 and N772). This can be seen by comparing the recovery slopes at the two ends of the STSA spectrum, where N220, with an average particle size of 20–25 nm [49,50], has a recovery rate three times faster than that of N772, with an average particle size of 71–96 nm [49,50]. Physical aging affects the mechanical, thermodynamic, and physical properties of the material [44] therefore, considering the equilibrium of the viscoelastic properties as the macroscopic display of the thermodynamic equilibrium of the structure, it could be inferred from Figure 7 that smaller particle sizes lead to a faster recovery of the thermodynamic equilibrium.

Figure 8 shows a weaker dependency between the storage and loss modulus slopes and the oil absorption number of the compressed sample. In this case, there is a maximum in the distribution of the three slopes against the COAN, indicating slower recoveries for those carbon black types at both ends of the spectrum. However, the shifting values obtained at the middle COAN range (N330, N220) suggest that further testing on additional carbon black types would be needed to derive a definite conclusion on this tendency.

The stronger dependency of the recovery rate on the STSA reinforces the idea of the physical aging process being dominated by the surface area rather than by the carbon black structure. The filler surface area has a direct influence on the gap distance between fillers and therefore the length of the stiffer glassy-like polymer bridges and the lower chain mobility close to the filler interface, governing the viscoelastic properties of the material. This reduction of the chain mobility in the filler–filler gap region results in a broadening of the glass transition range and a distribution of relaxation times [43]. Following this approach on the effect of the surface area together with the tendencies seen in Figure 7 and Figure 8, it seems plausible to infer that physical aging, a process directly related to the glass transition and the chains’ ability to rearrange toward equilibrium, will be itself also highly dependent on the particle surface area.

These experimental findings reveal the different effects of physical aging on the filled compound, leading to the contrasting evolution of the elastic and viscous parts of the modulus. This idea can be correlated to the results on the dynamic origin of filler networking [51], showing a combination of depletion interaction and excluded volume forces between the carbon black surface and the polymer chains. Also in this line, the results obtained from structural recovery test data [52,53,54,55] suggest the presence of different time scales on the physical aging process, as can be also seen here for the storage and loss modulus evolutions.

## 4. Conclusions

In this investigation, the torsion pendulum has been used to monitor the evolution of the dynamic properties of filler reinforced rubber after a temperature change. The goal being to characterize the physical aging process by means of its effect on the viscoelastic properties of the material, the storage and loss modulus. The obtained results show that the torsion pendulum has been able to successfully capture the effects of physical aging on the mechanical properties of the six tested compounds. The storage modulus shows an increase with physical aging time of up to 12.3% between its initial value and its value after 4 days, while the loss modulus presents a maximum decrease of 9.4%. The different tendencies of storage and loss modulus reflect the effect of physical aging on the rubber and filler components, coherent with literature results on the dynamic origins of filler networking and the time-dependent recovery behavior of the filler network. The results obtained after studying the dependency of the recovery rate of the properties and the carbon black surface area and structure seem to indicate a linear dependency with the first, and an imperceptible dependency on the latter. Furthermore, the shift observed in the mechanical properties from 10 min to 96 h ratifies the presence of physical aging at room temperatures for filler reinforced rubber and provides additional information about the separate evolution of the loss and storage modulus with physical aging time after a temperature change. Hence, the obtained data represent a step toward a more comprehensive characterization of physical aging and can therefore be used for a better prediction of the material performance in engineering applications as well as for the development of holistic constitutive models able to include the influence of physical aging on the material properties.

## Figures and Tables

**Table 1:** Material formulation of tested samples.

Component	Parts per Hundred (phr)
NR-SMR CV-60	100
Carbon Black	50
Zinc Oxide	5
Stearic Acid	3
Sulfur	2.5
TBBS ${}^{a}$	0.6

${}^{a}$ N-Tertiarybutyl-2-benzothiazole sulfenamide.

**Figure A2:**
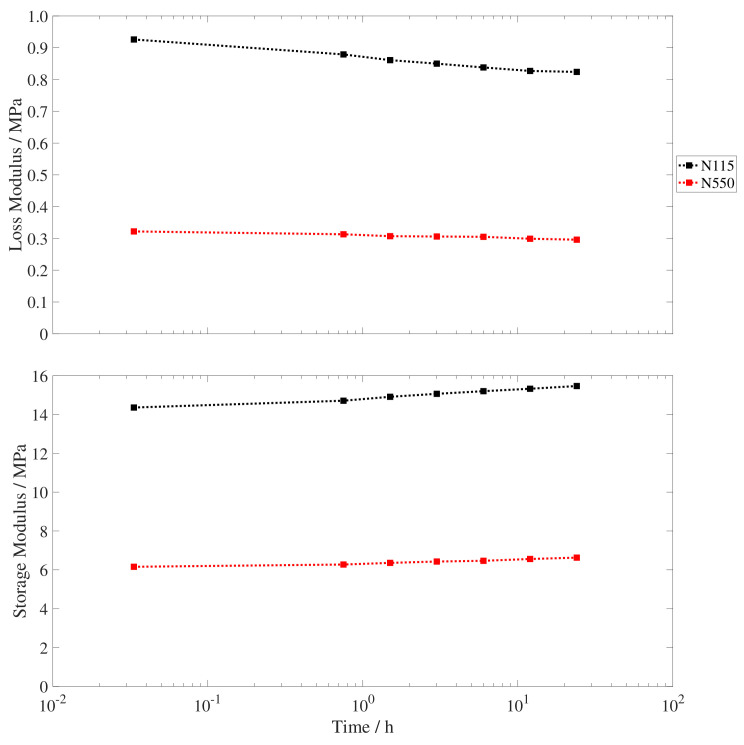
Evolution of the loss (top) and storage (bottom) modulus from 2 min to 48 h after a temperature drop from 70 to 20 °C. The different carbon black types are plotted in black (N115) and red (N550).

## Data Availability

Not applicable.

## Appendix A

**Table A1:** List of measurement times. At each measurement time, the sample is excited a minimum of 15 times, obtaining at least 15 repeats of the sinusoidal curve plotted in Figure 2 used to calculate the material properties.

Measurement times	10 min, 20 min, 30 min, 45 min, 60 min, 90 min,
	2 h, 3 h, 6 h, 9 h, 12 h, 24 h, 30 h, 36 h, 48 h, 54 h, 72 h, 96 h

## Appendix B. Comparison with DMA Data

The torsion pendulum results are now compared to Dynamic Mechanical Analysis data (DMA) obtained at Birla Carbon (Birla Carbon, Marietta, GA 30062, USA) for two different carbon black compounds, N115 and N550. The testing was performed using TA Instruments ARES G2 torsional rheometer with a dynamic strain amplitude level of 0.05%. The sample was annealed at 70 °C for 16 h and then quenched to 20 °C. The measurement times are 2 min, 45 min, 1.5 h, 3 h, 6 h, 12 h, 24 h. The DMA frequency sweep ranges from 0.01 to 10 Hz, and the values at 2.51 Hz are used here for easy visualization and comparison with torsion pendulum results. It can be seen from Figure A2 that the displayed trend is similar to that obtained with the torsion pendulum, showing an increasing storage modulus and a decreasing loss modulus with physical aging time. Additionally, the recovery rates from 45 min to 24 h are calculated for N550 to compare the evolution obtained with the two different methods, however, it is worth noting that the different temperature profiles of both experiments encumber a direct quantitative correlation. The storage modulus recovery rates are 0.014 MPa/h and 0.009 MPa/h for the DMA and torsion pendulum respectively. For the loss modulus, the recovery rates are $3.01\times 10^{-4}\;\mathrm{MPa}/h$ and $3.35\times 10^{-4}\;\mathrm{MPa}/h$ for the DMA and torsion pendulum procedures. The difference in the storage modulus recovery rate between the two methods may be due to the larger temperature drop implemented during the DMA testing. The values obtained for the loss modulus recovery rates are very close for both procedures, which may be explained by this rate being calculated on the slow range of the loss modulus recovery regime (>10 min), while a larger difference might be expected between the initial slopes calculated immediately after the temperature change.
